# What matters to families about the healthcare of preterm or low birth weight infants

**DOI:** 10.1093/eurpub/ckac129.389

**Published:** 2022-10-25

**Authors:** D Odd, M Mann, H Beetham, E Dorgeat, T Isaac, A Ashman, F Wood

**Affiliations:** 1 Division of Population Medicine, Cardiff University, Cardiff, UK; 2 Paediatrics Department, Yeovil District Hospital, Yeovil, UK; 3 Specialty Training Programme, Public Health Wales, Cardiff, UK

## Abstract

**Introduction:**

Preterm and low birth weight (LBW) infants have complex long-term healthcare needs. The impact on families of caring for a sick infant is increasingly understood, with consequences for attachment and bonding and parental health and wellbeing immediately after birth and beyond. In this qualitative evidence synthesis, we aimed to understand what matters to families about the care provided to preterm or LBW infants in hospital and the community.

**Methods:**

We searched nine databases and the reference lists of included studies for eligible studies using qualitative methods examining the views of families on healthcare for preterm or LBW infants. We used the Critical Appraisal Skills Programme checklist for qualitative studies to assess study quality and the GRADE-CERQual approach to assess confidence in each review finding. Studies were sampled after data saturation, and thematic synthesis techniques were used for analysis.

**Results:**

203 studies were eligible for inclusion. We selected 49 studies from 25 countries for the analysis, based on methodological quality, data richness and on ensuring representation from settings with varying resources. Eight analytical themes were identified. Confidence in most results was moderate to high. What mattered to carers was a positive outcome for the child; active involvement in care; support to cope at home after discharge; emotional support for the family; the healthcare environment; their information needs were met; logistical support was available; and positive relationships with staff.

**Conclusions:**

Enabling a positive post-natal period for families of small and sick infants is difficult. Experiences of care for preterm or LBW infants vary, but we found high consistency in what matters to families. This information can be used to shape global recommendations on support for infants and carers. More research is needed on what matters to parents who receive community-based care, especially in low resource settings.

**Key messages:**

• We found high consistency across settings in what matters to families in the care of preterm infants.

• Understanding carers views and values ensures that care can be planned to meet the needs of infants and families.

